# RAD51 135G>C substitution increases breast cancer risk in an ethnic-specific manner: a meta-analysis on 21236 cases and 19407 controls

**DOI:** 10.1038/srep11588

**Published:** 2015-06-25

**Authors:** Deepa Sekhar, Singh Pooja, Sandeep Kumar, Singh Rajender

**Affiliations:** 1 Division of Endocrinology, Central Drug Research Institute, Lucknow, India; 2 Department of Pathology, King George’s Medical University, Lucknow, India; 3 All India Institute of Medical Sciences, Bhopal, India

## Abstract

RAD51 is a homolog of bacterial RecA protein, which plays an important role in preserving stability of the genome. RAD51 interacts with BRCA1 and BRCA2 for homologous recombination repair. A functional polymorphism (135G > C) in the *RAD51* gene has been a subject of great interest, which is evidenced by at least 28 case-control studies and eight meta-analyses undertaken on this polymorphism till now. We undertook a meta-analysis on *RAD51* 135G > C data for 21236 cases and 19407 controls pooled from 28 studies on breast cancer in women. Pooled data analysis suggested a significant association of the substitution with breast cancer in the recessive model (GG + GC versus CC) and in the co-dominant models comparing GG versus CC and GC versus CC. Analysis of the results suggested that ‘CC’ genotype is a significant breast cancer risk factor in comparison to ‘GG’ and ‘GC’ genotypes. We also undertook pooled analyses on different ethnic groups and found that ‘CC’ was a strong risk factor in Caucasians, but not in East-Asians and populations of mixed ethnicity. In conclusion, the *RAD51* 135G > C substitution in the homozygous form (CC) increases the risk of breast cancer in an ethnic-specific manner.

Breast cancer (BC) is the most prevalent cancer amongst women worldwide. It represents high incidence rates in North America and Western European countries in comparison to Asian or African populations^1^. The mechanism leading to breast cancer is not yet known, but it is a disorder caused by a number of genetic or environmental factors such as family history, multiparity, early menarche, age at first childbirth and late menopause^2^. Linkage mapping, genetic association studies, and recent GWAS studies have provided insights into the contribution of genetic factors to breast cancer risk. The major breast cancer susceptibility genes, *BRCA1* and *BRCA2*, play a role in the homologous recombination pathway (HR) for repairing the double strand breaks (DSB) in DNA^3^. Genetic instability caused by deficient double strand break repair is considered an important factor in the onset of breast cancer^4^. It could modify the repair capacity leading to accumulation of mutations and hence, cancer. It is a well-known fact that such damage can be easily caused by chemicals, radiations and other factors which could enhance the risk of malignancies^5^.

RAD51 is a homolog of bacterial RecA protein, which plays an important role in preserving stability of the genome^6^. It is located on chromosome 15q15.1 in humans and acts as a core element in the homolog-dependent recombinational repair of the double strand breaks through assembled nucleoprotein filaments on single stranded DNA^7^. It in turn mediates the strand invasion and exchange between the homologous DNA and damaged site^8^. RAD51 interacts with BRCA1 and BRCA2 for homologous recombination repair as it is a major requirement for mitotic/meiotic recombination^9^. A functional polymorphism in the *RAD51* gene, changing a guanine to cytosine at position 135 in its 5’ untranslated region, has been accused of modulating breast cancer risk by altering gene transcription^8^. The 135G > C polymorphism affects mRNA stability and in turn translation efficiency. It thus produces altered products, which further influence the functionality of a multi-protein DNA repair complex consisting of BRCA1, BRCA2 and RAD51^10^.

Many epidemiological studies have been carried out to examine the association between *RAD51* 135G > C polymorphism and the risk of breast cancer in different populations. But, inconsistent results, even within the same population, have hampered the consensus building regarding the impact of this polymorphism on breast cancer risk. For example, Sliwinski *et al.* did not find any association between *RAD51* 135G > C polymorphism and breast cancer risk, which Synoweic *et al.* found in a Polish population^10^,^11^. Inconsistency across the studies may be due a large number of factors, including study design, criteria for recruitment of cases and controls, statistical tests applied, and above all ethnicities of study populations. Meta-analysis is a powerful approach for data pooling and analysis to reach consensus, despite the heterogeneity across studies. We undertook the present study to assess the impact of the *RAD51* 135G > C polymorphism on breast cancer risk.

## Results

### Eligible studies

**Figure 1:**
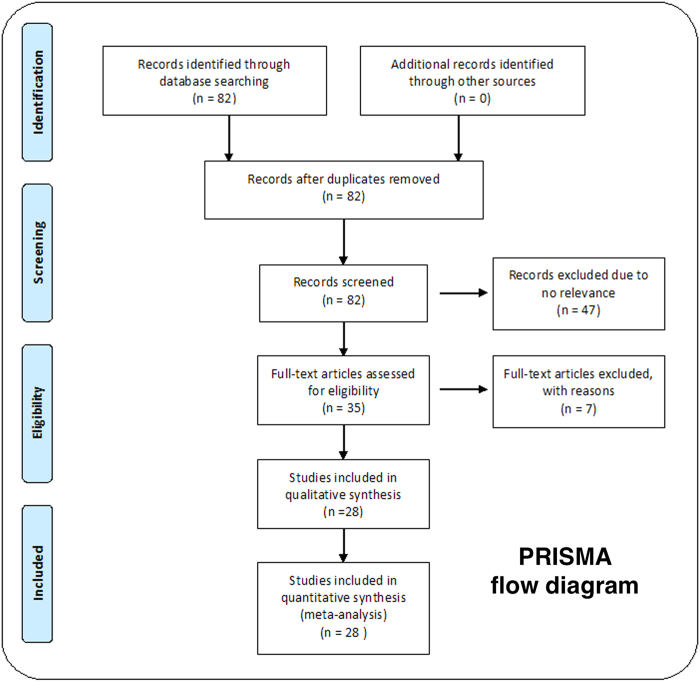
PRISMA flow diagram showing the total number of hits followed by short-listing of studies for inclusion in the meta-analysis.

Eighty-two studies were retrieved as a result of a literature search. Forty-seven were excluded as they were not relevant to the aim of our study (association of *RAD51* 135G > C with breast cancer). A total of thirty-five studies were then considered for inclusion. Seven studies were excluded as they either lacked data on *RAD51* 135G > C substitution^3^,^12^,^13^,^14^,^15^,^16^ or were not case-control studies^17^. Hence, a total of 28 case-control studies (21236 cases and 19407 controls) following a strict exclusion-inclusion criteria were included in the meta-analysis (Fig. 1). The main characteristics of these studies are depicted in the supplementary table S1. There were eighteen studies on Caucasians, four on East-Asians and six on mixed populations. All the cases involved in these studies were pathologically confirmed and age-matched controls were recruited from healthy populations.

### Pooled analysis

**Figure 2:**
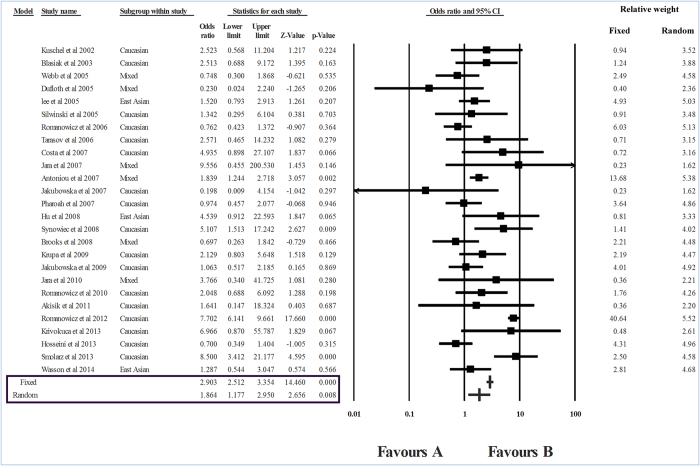
Forest plot showing the odds ratio, p value and direction of association between RAD51 135G > C polymorphism and breast cancer. The Z value shows the degree and direction of the relationship, wheras the P value shows the significance of the relationship. The horizontal bar shows the range of OR with a square in the centre, the size of the latter is directly proportional to the weight given to each study. The direction of projection of the horizontal bar shows the direction of association.

The meta-analysis results have been summarized in Table 1. There was significant heterogeneity across all levels of analysis; which justified the choice of the random effects model of analysis. However, we have presented results of both fixed effects and random effects models of analysis. Further, we have conducted analyses using all genetic models to properly characterize the association between *RAD51* 135G > C substitution and BC risk. In the dominant model of analysis, we found no association between *RAD51* 135G > C polymorphism and BC risk (OR = 1.033, P = 0.578). However, the recessive model of analysis, (GG + GC) vs. CC, showed significant association between the polymorphism and BC risk (I^2^ = 85.28, P_heterogeneity_ = 0, OR = 1.864, P = 0.008) (Fig. 2). In the co-dominant model (GG vs. CC), differential distribution of genotypes showed a significant association with BC risk (I^2^ =  68.83, P_heterogeneity_ = 0, OR = 1.671, P = 0.004). Another co-dominant model, GC vs. CC also showed an association of *RAD*51 135G > C polymorphism with BC risk (I^2^ = 88.24, P_heterogeneity_ = 0, OR = 1.984, P = 0.013). It can be concluded from the above that double substitution (CC) is a highly significant risk factor against homozygous common (GG) and heterozygous genotypes (GC); however, heterozygous does not appear to be a significant risk factor against homozygous common genotype.

### Pooled analysis on the basis of ethnicity

Since there was significant heterogeneity in the outcome and the inference of the studies, we undertook further analysis on groups based on ethnicity. We stratified all studies into three groups depending upon their ethnic affinity. ‘Mixed’ groups included studies where the authors had mentioned the mixed nature of study samples. Pooled analysis of each group was undertaken using all genetic models of analysis: dominant, recessive, and co-dominant.

**Figure 3:**
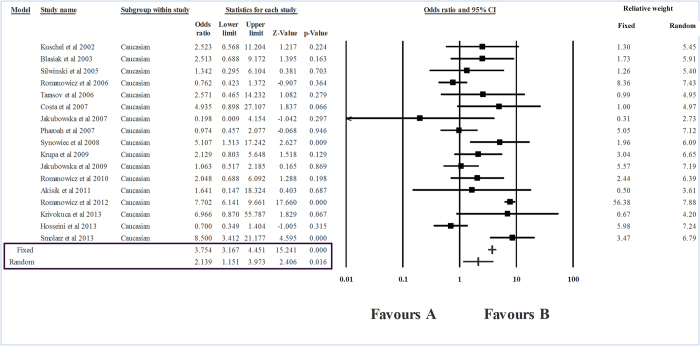
Forest plot showing the odds ratio, p value and direction of association between RAD51 135G > C polymorphism and breast cancer in Caucasian sub-group.

**Figure 4:**
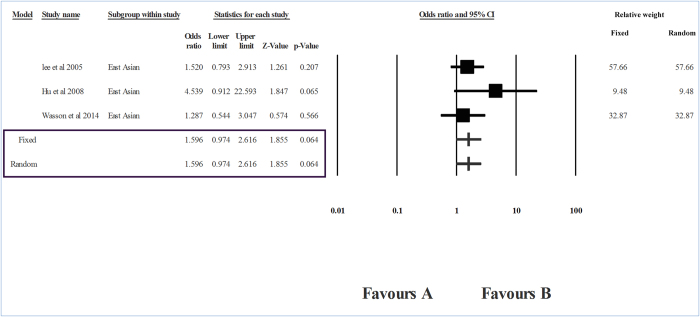
Forest plot showing the odds ratio, p value and direction of association between RAD51 135G > C polymorphism and breast cancer in East Asians sub-group.

**Figure 5:**
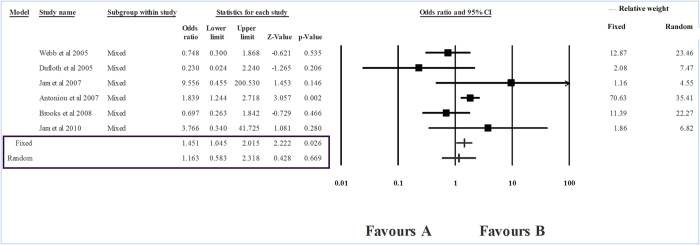
Forest plot showing the odds ratio, p value and direction of association between RAD51 135G > C polymorphism and breast cancer in populations of mixed ethnicity.

The level of heterogeneity in the sub-group analysis was high; therefore, we used the random effects model of analysis. The group-wise analysis showed a strong association between *RAD51* CC genotype and breast cancer risk in Caucasians (I^2^ = 87.32, P_heterogeneity_ = 0, OR    = 2.139, P = 0.016) (Table 2, Fig. 3). However, in the case of East Asians, no association between *RAD*51 genotypes and breast cancer was seen (P = 0.064) (Fig. 4). In the ‘mixed’ sub-group, the substitution was unrelated to the risk of breast cancer (P = 0.669) (Table 2, Fig. 5).

### Sensitivity analysis

**Figure 6:**
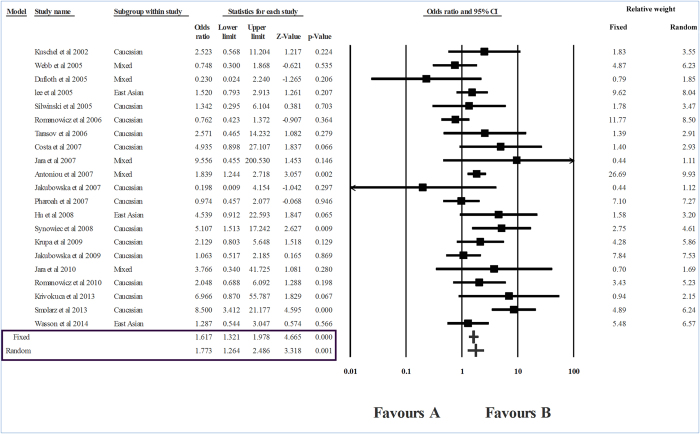
Forest plot upon sensitivity analysis based on exclusion of the studies deviating from the Hardy Weinberg equilibrium.

Since all the studies were not carried out using equally stringent protocols, some of them may bias the results in the pooled analysis. Therefore, a sensitivity analysis was carried out to identify the studies that could have significantly biased the overall conclusion. We analyzed the control data of each study for fitness in the Hardy Weinberg equilibrium. Seven studies did not comply with the Hardy Weinberg equilibrium; therefore, a pooled analysis was carried out after exclusion of these. Analysis on the remaining twenty-one studies showed that the overall inference was not significantly biased by the studies not following the Hardy Weinberg equilibrium. Thus, the conclusion that ‘CC’ genotype increased BC risk as compared to its other variants, GG or GC (I^2^ = 51.94, P_heterogeneity_ = 0, OR = 1.773, P = 0), stood firm (Fig. 6). We found none of the studies to be sensitive enough to strongly bias the overall conclusion of this meta-analysis.

### Publication bias

**Figure 7:**
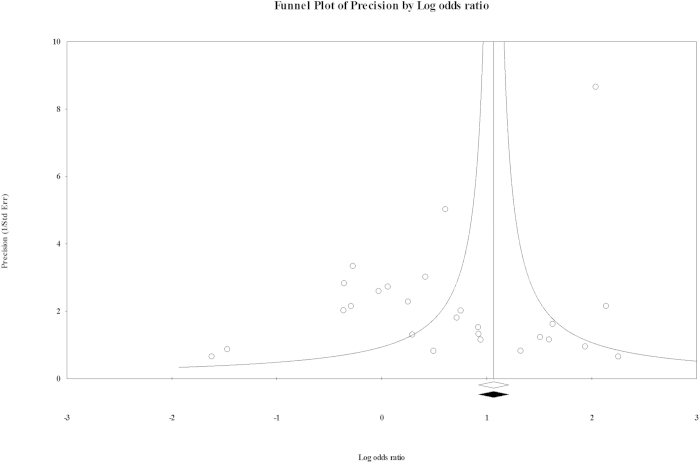
Funnel plot for publication bias presenting a symmetric distribution of studies on either side of the funnel.

Begg’s funnel plot and Egger’s regression intercept tests were performed to calculate the publication bias. However, both these tests did not provide any evidence of publication bias. The shapes of the plots ruled out the presence of asymmetry in the overall analysis, which was confirmed by the Egger’s test (P = 0.439). Classic fail safe ‘N’ was 28 (P = 0.460, Z = 0.738), suggesting that an addition of 28 null studies would be needed to bring the P value to a non-significant range. In the same manner, Orwin’s fail safe ‘N’ was 32, suggesting that the number of studies required to bring the observed odds ratio over 0.999 would be 32. Since the chances of missing so many studies for twenty-eight pooled studies are negligible, we concluded that the results are stable and the pooled analysis was not biased. Our observation of symmetric funnel plots and non-significant statistical tests confirmed the absence of bias (Fig. 7).

## Discussion

Meta-analysis is a tool to develop consensus when the results across the case-control studies vary. However, it is interesting to note that the *RAD51* 135G > C polymorphism has been subjected to meta-analyses eight times, but with different outcomes. This has led to an interesting scenario of variation across the case-control studies and pooled analyses. Interestingly, similar to the issue with the case-control studies, the inference in the meta-analyses on *RAD51* 135G > C varies from one extreme of association to another extreme of no association. Yu *et al.* conducted a meta-analysis on twelve studies (7065 cases and 6981 controls) and suggested that *RAD51* 135G > C might not modify BC risk in non- *BRCA1* and *BRCA2* mutation carriers^9^. Another meta-analysis by Wang *et al.* on nine studies (13,241 cases and 13,203 controls) suggested an association of this polymorphism with breast cancer risk^8^. The results of a sensitivity analysis suggested that women with CC genotype had a higher risk of breast cancer in comparison to those with GG or GC genotypes^8^. In strong contrast to both the above, Sun *et al.* performed a meta-analysis on seventeen studies (12,153 cases and 10,245 controls) and found that *RAD51* 135 G> C polymorphism may be a protective factor against breast cancer^4^. Zhou *et al.* conducted a meta-analysis on fourteen studies (12,183 cases and 10,183 controls) and observed that *RAD51* variant 135C homozygote is associated with an elevated risk of breast cancer among *BRCA2* mutation carriers, but not in *BRCA1* mutation carriers^18^. Gao *et al.* conducted a meta-analysis on twenty-one studies, including 12,860 cases and 11,169 controls and observed that CC genotype was associated with a high risk of breast cancer as compared to its other variants^7^.

The present meta-analysis included 21236 cases and 19407 controls from twenty-eight studies on *RAD51* 135G > C polymorphism and breast cancer risk. We used robust methods and undertook a sensitivity analysis by excluding the studies that deviated from the HW equillibrium. The results suggested that women carrying CC genotypes are at an increased risk of breast cancer as compared to those with other genotypes (GG or GC). On the basis of available biological evidence, the plausible mechanism of increased risk is its effect on the stability of mRNA and in turn the efficiency of translation. The production of altered protein as a result of this mutation influences the functionality of a multi-protein DNA repair complex consisting of BRCA1, BRCA2 and RAD51^18^. Thus, compromised DNA repair system as a result of faulty RAD51 protein increases breast cancer risk.

Literature review suggests that there are contradictory conclusions regarding the association of *RAD51* 135G > C polymorphism with the risk of breast cancer. Brooks *et al.* observed that white women were more susceptible to the disease as compared to Asian women (P <0.0001). 37.4% of the black women had at least one copy of the variant allele *RAD51* 135C while among non-Jewish white, Jewish white and other ethnic populations, the frequencies were 15.9%, 9.6% and 17.3%, respectively^19^. In a study on an Indian population of East-Asian ethnicity, Wasson *et al.* observed that genetic contrast in cell cycle and DNA repair genes actively contributed to the risk of breast cancer. A group of betel quid chewing cases showed an elevated risk of cancer, which was linked to betel quid carcinogens, minor alleles of *BRCA2* mutation and C allele of the *RAD51* gene^1^. In contrast, Blasiak *et al.* suggested that in Caucasians, the G/C polymorphism of the *RAD51* gene may not directly correlate with the progression of the disease and hence, it is not a useful marker^20^. Dufloth *et al.* found no statistically significant difference in the genotype frequency of *RAD51* ‘C’ between cases and controls in a population of mixed ethnicity (P = 0.96)^21^. We also conducted a sub-group analysis on three ethnic groups: Caucasians, East Asians, and mixed. We found that ethnicity of the study population affected the association of *RAD51* 135G > C polymorphism with the risk of breast cancer significantly. The substitution associated with breast cancer in Caucasians, but not in East Asians and populations (study groups) having mixed ethnicity.

In conclusion, homozygous substitution (CC) at the *RAD51* 135G > C locus increases the risk of breast cancer significantly. Nevertheless, heterozygous substitution may not raise the risk considerably. Further, the risk is strongly affected by ethnicity of the study population, as group-wise analysis suggested strong impact of this polymorphism in Caucasians, but not in East Asians and populations of mixed ethnicities. A large sample size and the absence of publication bias further strengthened our conclusions. We conclude that *RAD51* 135G > C substitution may serve as a useful marker for screening of breast cancer risk; Nevertheless, its use may be restricted to the Caucasian populations. Since about 65% of the studies included in this meta-analysis were undertaken on Caucasian subjects, further studies on East Asians may be required to confidently rule out the association of *RAD51* 135G > C substitution with breast cancer in these populations. Since in the case of mixed ethnicity groups, one of the analysis models (fixed effect) suggested a significant correlation, reporting of genotype details ethnicity-wise is encouraged to help pooled analysis reach appropriate conclusions.

## Methods

### Literature search

Relevant studies were chosen by searching ‘Google Scholar (scholar.google.co.in)’ ‘ScienceDirect (www.sciencedirect.com)’ and ‘Pubmed (www.pubmed.com)’ databases up to the 31^st^ of October 2014 as the publication date, using the following keywords: ‘RAD 51’, ‘polymorphism’ and ‘breast cancer’ in various combinations. All the studies were checked for their references for identification of other relevant studies. The studies published in English only were considered for further screening. We did not specify a minimum sample size as the criterion for inclusion of a study in the analysis. In the case of multiple studies from a research group, the study with the largest sample size was chosen to avoid over-representation or duplication of data.

### Data extraction

The data against the following variables were extracted from each study; first author’s name, year of publication, ethnicity of subjects, source of the samples, and genotypes of cases and controls. To avoid errors in the pooled analysis, the data extraction was performed by DS and SR, independently.

### Inclusion and exclusion criteria

The hits obtained as a result of the literature search were subjected to the following inclusion/exclusion criteria to select the studies for pooled analysis;

The inclusion criteria comprised of the following: i) The studies looking for correlation of *RAD51* 135G > C substitution with BC risk. ii) Each study was an independent case-control study. iii) The statistical methods and the purpose of all the studies were similar. iv) The given information was enough to calculate the odds ratio. v) SNP genotyping had been undertaken using standard genotyping techniques. vi) Patients within the study had been recruited in accordance with the standard diagnostic parameters.

The exclusion criteria included: i) The study was not a case-control study. ii) The study did not aim to look for correlation of *RAD51* 135G > C substitution with breast cancer risk. iii) The study had reviewed the literature and not presented new data. iv) The raw data were unavailable in the article and the authors did not respond to three requests by e-mail. v) The study had been expanded to include more number of samples at a later stage.

### Statistical analysis

The effectiveness of the association between *RAD51* 135G > C polymorphism and breast cancer risk was evaluated by odds ratio (OR) with the corresponding 95% confidence interval (CI). The pooled OR is computed by the fixed effects model (the Mantel- Haenszel method) when there was a lack of heterogeneity between studies, otherwise, the random effects model (the Der Simonian and Laird method) is preferred^22^,^23^. Since the meta-analysis pools data from studies conducted by different people across the world, we had a priori preference to use the random effects model, which is more stringent and less likely to favor an odd observation, unless there is a real effect. Heterogeneity was examined by the chi-square based Q test and P values  > 0.05 were taken to suggest the lack of heterogeneity across the studies^24^,^25^,^26^,^27^,^28^,^29^,^30^,^31^,^32^,^33^,^34^,^35^,^36^,^37^,^38^,^39^,^40^,^41^,^42^,^43^.

The OR and 95% CI were calculated using different genetic models: dominant, co-dominant and recessive. Bonferroni’s correction for multiple testing was not considered given a limited number of tests that were pre-hypothesized. Stratification based on ethnicity (Caucasians, East Asians and mixed) was employed for group-wise analyses. Genotype data of the control groups were studied for fitness in the Hardy Weinberg Equilibrium (HWE). Publication bias was assessed using Egger’s linear regression test that was followed by visual inspection of the funnel plot. All statistical tests were performed using the Comprehensive Meta Analysis software (version 2).

## Additional Information

**How to cite this article**: Sekhar, D. *et al.* RAD51 135G > C substitution increases breast cancer risk in an ethnic-specific manner: a meta-analysis on 21236 cases and 19407 controls. *Sci. Rep.* **5**, 11588; doi: 10.1038/srep11588 (2015).

## Supplementary Material

Supplementary Table S1

## Figures and Tables

**Table 1:** Results of meta-analysis using different genetic models.

Analysis models	Heterogeneity		Fixed		Random		Inference
	I^2^	P	OR	P	OR	P	
GG vs. (GC + CC)	67.50	0	0.998	0.938	1.033	0.578	Not associated
GG vs. GC	80.07	0	0.912	0.001	0.907	0.210	Not associated
GG vs. CC	68.83	0	2.051	0	1.671	0.004*	Significant association
GC vs. CC	88.24	0	3.143	0	1.984	0.013*	Significant association
(GG + GC) vs. CC	85.28	0	2.903	0	1.864	0.008*	Significant association

^*^Indicates statistical significance.

**Table 2:** Results of sub-group meta-analysis using different genetic models.

Analysis models	Heterogeneity		Fixed		Random		Inference
	I^2^	P	OR	P	OR	P	
**Caucasians**							
**GG vs. (GC + CC)**	77.15	0	1.011	0.797	0.989	0.911	Not significant
**GG vs. GC**	86.11	0	0.838	0	0.793	0.088	Not significant
**GG vs. CC**	73.54	0	2.398	0	1.852	0.011*	Significant association
**GC vs. CC**	90.2	0	4.408	0	2.388	0.023*	Significant association
**(GG + GC) vs. CC**	87.32	0	3.754	0	2.139	0.016*	Significant association
**East–Asians**							
**GG vs. (GC + CC)**	16.04	0.31	1.095	0.314	1.112	0.295	Not significant
**GG vs. GC**	27.49	0.25	1.049	0.607	1.074	0.535	Not significant
**GG vs. CC**	0	0.37	1.611	0.061	1.611	0.061	Not significant
**GC vs. CC**	0	0.41	1.641	0.061	1.641	0.061	Not significant
**(GG + GC) vs. CC**	0	0.39	1.596	0.064	1.596	0.064	Not significant
**Mixed**							
**GG vs. (GC + CC)**	0	0.63	0.97	0.431	0.97	0.431	Not significant
**GG vs. GC**	0	0.48	0.948	0.186	0.948	0.186	Not significant
**GG vs. CC**	49.52	0.08	1.443	0.029	1.169	0.652	Not significant
**GC vs. CC**	56.7	0.04	1.513	0.016	1.129	0.754	Not significant
**(GG + GC) vs. CC**	51.01	0.07	1.451	0.026	1.163	0.669	Not significant

^*^Indicates statistical significance

## References

[b1] Wasson M. K. *et al.* Association of DNA repair and cell cycle gene variations with breast cancer risk in Northeast Indian population: a multiple interaction analysis. Tumour Biol. 35, 5885–5894 (2014). 24604328 10.1007/s13277-014-1779-2

[b2] Costa S. *et al.* DNA repair polymorphisms might contribute differentially on familial and sporadic breast cancer susceptibility: a study on a Portuguese population. Breast Cancer Res Treat. 103, 209–217 (2007). 17063276 10.1007/s10549-006-9364-z

[b3] Ding S. L. *et al.* Genetic variants of BLM interact with RAD51 to increase breast cancer susceptibility. Carcinogenesis 30, 43–49 (2009). 18974064 10.1093/carcin/bgn233

[b4] Sun H. *et al.* RAD51 G135C polymorphism is associated with breast cancer susceptibility: a meta-analysis involving 22,399 subjects. Breast Cancer Res Treat. 125, 157–161 (2011). 20454923 10.1007/s10549-010-0922-z

[b5] Zhang B. B., Wang D. G., Xuan C., Sun G. L. & Deng K. F. Genetic 135G/C polymorphism of RAD51 gene and risk of cancer: a meta-analysis of 28,956 cases and 28,372 controls. Fam Cancer. 13, 515–526 (2014). 24859942 10.1007/s10689-014-9729-0

[b6] Romanowicz M. H., Smolarz B., Zadrozny M. & Kulig A. Analysis of RAD51 polymorphism and BRCA1 mutations in Polish women with breast cancer. Exp Oncol. 28, 156–159 (2006). 16837909

[b7] Gao L. B. *et al.* RAD51 135G/C polymorphism and breast cancer risk: a meta-analysis from 21 studies. Breast Cancer Res Treat. 125, 827–835 (2011). 20640595 10.1007/s10549-010-0995-8

[b8] Wang Z., Dong H., Fu Y. & Ding H. RAD51 135G > C polymorphism contributes to breast cancer susceptibility: a meta-analysis involving 26,444 subjects. Breast Cancer Res Treat. 124, 765–769 (2010). 10.1007/s10549-010-0885-0 20396943

[b9] Yu K. D., Yang C., Fan L., Chen A. X. & Shao Z. M. RAD51 135G > C does not modify breast cancer risk in non-BRCA1/2 mutation carriers: evidence from a meta-analysis of 12 studies. Breast Cancer Res Treat. 126, 365–371 (2011). 10.1007/s10549-010-0937-5 20461453

[b10] Sliwinski T. *et al.* Polymorphisms of the BRCA2 and RAD51 genes in breast cancer. Breast Cancer Res Treat. 94, 105–109 (2005). 16261408 10.1007/s10549-005-0672-5

[b11] Synowiec E., Stefanska J., Morawiec Z., Blasiak J. & Wozniak K. Association between DNA damage, DNA repair genes variability and clinical characteristics in breast cancer patients. Mutat Res. 648, 65–72 (2008). 18977234 10.1016/j.mrfmmm.2008.09.014

[b12] Sassi A., Popielarski M., Synowiec E., Morawiec Z. & Wozniak K. BLM and RAD51 genes polymorphism and susceptibility to breast cancer. Pathol Oncol Res. 19, 451–459 (2013). 23404160 10.1007/s12253-013-9602-8 PMC3708281

[b13] Le Calvez-Kelm F. *et al.* RAD51 and breast cancer susceptibility: no evidence for rare variant association in the Breast Cancer Family Registry study. PLoS One. 7, e52374 (2012). 23300655 10.1371/journal.pone.0052374 PMC3531476

[b14] Ricks-Santi L. J. *et al.* Association of Rad51 polymorphism with DNA repair in BRCA1 mutation carriers and sporadic breast cancer risk. BMC Cancer. 11, 278 (2011). 21708019 10.1186/1471-2407-11-278 PMC3146938

[b15] Silva S. N. *et al.* Breast cancer risk and common single nucleotide polymorphisms in homologous recombination DNA repair pathway genes XRCC2, XRCC3, NBS1 and RAD51. Cancer Epidemiol. 34, 85–92 (2010). 20004634 10.1016/j.canep.2009.11.002

[b16] Barroso E. *et al.* FANCD2 associated with sporadic breast cancer risk. Carcinogenesis. 27, 1930–1937 (2006). 16679306 10.1093/carcin/bgl062

[b17] Kato M. *et al.* Identification of Rad51 alteration in patients with bilateral breast cancer. J Hum Genet. 45, 133–137 (2000). 10807537 10.1007/s100380050199

[b18] Zhou G. W., Hu J., Peng X. D. & Li Q. RAD51 135G > C polymorphism and breast cancer risk: a meta-analysis. Breast Cancer Res Treat. 125, 529–535 (2011). 10.1007/s10549-010-1031-8 20623332

[b19] Brooks J. *et al.* Polymorphisms in RAD51, XRCC2, and XRCC3 are not related to breast cancer risk. Cancer Epidemiol Biomarkers Prev. 17, 1016–1019 (2008). 18398049 10.1158/1055-9965.EPI-08-0065

[b20] Blasiak J. *et al.* Analysis of the G/C polymorphism in the 5’-untranslated region of the RAD51 gene in breast cancer. Acta Biochim Pol. 50, 249–253 (2003). 12673366

[b21] Dufloth R. M., Costa S., Schmitt F. & Zeferino L. C. DNA repair gene polymorphisms and susceptibility to familial breast cancer in a group of patients from Campinas, Brazil. Genet Mol Res. 4, 771–782 (2005). 16475125

[b22] Mantel N. & Haenszel W. Statistical aspects of the analysis of data from retrospective studies of disease. J Natl Cancer Inst. 22, 719–748 (1959). 13655060

[b23] DerSimonian R. & Laird N. Meta-analysis in clinical trials. Control Clin Trials. 7, 177–188 (1986). 3802833 10.1016/0197-2456(86)90046-2

[b24] Kuschel B. *et al.* Variants in DNA double-strand break repair genes and breast cancer susceptibility. Hum Mol Genet. 11, 1399–1407 (2002). 12023982 10.1093/hmg/11.12.1399

[b25] Kadouri L. *et al.* A single-nucleotide polymorphism in the RAD51 gene modifies breast cancer risk in BRCA2 carriers, but not in BRCA1 carriers or non-carriers. Br J Cancer. 90, 2002–2005 (2004). 15138485 10.1038/sj.bjc.6601837 PMC2409456

[b26] Webb P. M. *et al.* Double-strand break repair gene polymorphisms and risk of breast or ovarian cancer. Cancer Epidemiol Biomarkers Prev. 14, 319–323 (2005). 15734952 10.1158/1055-9965.EPI-04-0335

[b27] Lee K. M. *et al.* Genetic polymorphisms of selected DNA repair genes, estrogen and progesterone receptor status, and breast cancer risk. Clin Cancer Res. 11, 4620–4626 (2005). 15958648 10.1158/1078-0432.CCR-04-2534

[b28] Tarasov V. A. *et al.* Genetically determined subdivision of human populations with respect to the risk of breast cancer in women. Dokl Biol Sci. 406, 66–69 (2006). 16572816 10.1134/s0012496606010182

[b29] Chang T. W. *et al.* Glutathione S-transferase polymorphisms associated with risk of breast cancer in southern Taiwan. Breast. 15, 754–761 (2006). 16713266 10.1016/j.breast.2006.03.008

[b30] Jara L. *et al.* RAD51 135G >C polymorphism and risk of familial breast cancer in a South American population. Cancer Genet Cytogenet. 178, 65–69 (2007). 10.1016/j.cancergencyto.2007.05.024 17889711

[b31] Antoniou A. C. *et al.* RAD51 135G > C modifies breast cancer risk among BRCA2 mutation carriers: results from a combined analysis of 19 studies. Am J Hum Genet. 81, 1186–1200 (2007). 17999359 10.1086/522611 PMC2276351

[b32] Jakubowska A. *et al.* The RAD51 135G > C polymorphism modifies breast cancer and ovarian cancer risk in polish BRCA1 mutation carriers. Cancer Epidemiol Biomarkers Prev. 16, 270–275 (2007). 10.1158/1055-9965.EPI-06-0562 17301259

[b33] Pharoah P. D., Tyrer J., Dunning A. M., Easton D. F. & Ponder B. A. Association between common variation in 120 candidate genes and breast cancer risk. PLoS Genet. 3, 401–406 (2007). 10.1371/journal.pgen.0030042 PMC1828694 17367212

[b34] Hu R. *et al.* Association of polymorphisms of N372H in BRCA2 gene and 135G/C in RAD51 gene and breast cancers. Sichuan Da Xue Xue Bao Yi Xue Ban. 39, 973–975 (2008). 19253839

[b35] Krupa R. *et al.* Polymorphism of the homologous recombination repair genes RAD51 and XRCC3 in breast cancer. Exp Mol Pathol. 87, 32–35 (2009). 19426727 10.1016/j.yexmp.2009.04.005

[b36] Jakubowska A. *et al.* Do BRCA1 modifiers also affect the risk of breast cancer in non-carriers? Eur J Cancer. 45, 837–842 (2009). 19071013 10.1016/j.ejca.2008.10.021

[b37] Jara L. *et al.* Variants in DNA double-strand break repair genes and risk of familial breast cancer in a South American population. Breast Cancer Res Treat. 122, 813–822 (2010). 20054644 10.1007/s10549-009-0709-2

[b38] Romanowicz H., Smolarz B., Baszczynski J., Zadrozny M. & Kulig A. Genetics polymorphism in DNA repair genes by base excision repair pathway (XRCC1) and homologous recombination (XRCC2 and RAD51) and the risk of breast carcinoma in the Polish population. Pol J Pathol. 61, 206–212 (2010). 21290343

[b39] Akisik E., Yazici H. & Dalay N. ARLTS1, MDM2 and RAD51 gene variations are associated with familial breast cancer. Mol Biol Rep. 38, 343–348 (2011). 20358297 10.1007/s11033-010-0113-3

[b40] Romanowicz H. *et al.* The association between polymorphisms of the RAD51-G135C, XRCC2-Arg188His and XRCC3-Thr241Met genes and clinicopathologic features in breast cancer in Poland. Eur J Gynaecol Oncol. 33, 145–150 (2012). 22611952

[b41] Krivokuca A. M. *et al.* RAD51 135G > C and TP53 Arg72Pro polymorphisms and susceptibility to breast cancer in Serbian women. Fam Cancer. 13, 173–180 (2014). 24114315 10.1007/s10689-013-9690-3

[b42] Hosseini M., Houshmand M. & Ebrahimi A. RAD51 polymorphisms and breast cancer risk. Mol Biol Rep. 40, 665–668 (2013). 23065228 10.1007/s11033-012-2105-y

[b43] Smolarz B. *et al.* RAD51 genotype and triple-negative breast cancer (TNBC) risk in Polish women. Pol J Pathol. 64, 39–43 (2013). 23625599 10.5114/pjp.2013.34602

