# Supplementary material for: RAD51 135G>C substitution increases breast cancer risk in an ethnic-specific manner: a meta-analysis on 21236 cases and 19407 controls
Source: Sci Rep. 2015 Jun 25;5:11588. doi: 10.1038/srep11588 (PMC4479800; doi:10.1038/srep11588)
Supplement: Supplementary Table S1 [file srep11588-s1.pdf]

RAD51 135G>C substitution increases breast cancer risk in an ethnic-specific manner: a meta-analysis on 21236 cases and 19407 controls

Deepa Sekhar[1], Singh Pooja[2], Sandeep Kumar[3], Singh Rajender[1]

Supplementary table S1: Characteristics and genotype data of the case-control studies included in the meta-analysis

No.	Author's name	Year	Country	Ethnicity	Source	GG	GC	CC	Total	GG	GC	CC	Total	P for HW equilibrium
1	Kuschel et al[24]	2002	UK	Caucasian	Population based	1904	255	13	2172	722	116	2	840	0.2344
2	Blasiak et al[20]	2003	Poland	Caucasian	Hospital based	11	28	7	46	21	35	4	60	0.0375*
3	Kadouri et al[25]	2004	UK	Caucasian	Hospital based	290	0	0	333	231	0	0	261	0*
4	Webb et al[26]	2005	Australia	Mixed	Population based	1221	212	11	1444	676	104	8	788	0.0822
5	Dufloth et al[21]	2005	Brazil	Mixed	Hospital based	144	24	1	169	103	13	3	119	0.0051
6	Lee et al[27]	2005	Korea	East-Asian	Hospital based	611	143	28	782	450	123	14	587	0.1142
7	Silwinski et al[10]	2005	Poland	Caucasian	Not reported	108	38	4	150	106	41	3	150	0.6735
8	Romanowicz et al[6]	2006	Poland	Caucasian	Hospital based	31	40	29	100	21	48	37	106	0.4509
9	Tarasov et al[28]	2006	Russia	Caucasian	Not reported	111	36	4	151	148	41	2	191	0.6505
10	Chang et al[29]	2006	Taiwan	East Asian	Hospital based	116	73	0	189	284	137	0	421	0*
11	Costa et al[2]	2007	Portugal	Caucasian	Hospital based	216	45	4	265	558	86	2	646	0.4909
12	Jara et al[30]	2007	Chile	Mixed	Population based	113	16	2	131	222	25	0	247	0.4021
13	Antoniou et al[31]	2007	UK	Mixed	Not reported	7683	1134	76	8893	6977	1130	38	8145	0.283
14	Jakubowska et al[32]	2007	Poland	Caucasian	Hospital based	210	48	0	258	188	68	2	258	0.1169
15	Pharoah et al[33]	2007	Multiple	Caucasian	Population based	1911	236	13	2160	1995	257	14	2266	0.0724
16	Hu et al[34]	2008	China	East Asian	Not reported	35	29	7	71	49	34	2	85	0.1602
17	Synowiec et al[11]	2008	Poland	Caucasian	Population based	18	10	13	41	17	27	4	48	0.138
18	Brooks et al[19]	2008	USA	Mixed	Population based	516	88	7	611	513	88	10	611	0.0086*
19	Krupa et al[35]	2009	Poland	Caucasian	Hospital based	91	33	11	135	105	63	7	175	0.5172
20	Jakubowska et al[36]	2009	Poland	Caucasian	Hospital based	785	207	15	1007	822	232	15	1069	0.7644
21	Jara et al[37]	2010	Chile	Mixed	Population based	232	33	2	267	441	58	1	500	0.5258

22	Romanowicz et al[38]	2010	Poland	Caucasian	Hospital based	141	69	10	220	157	58	5	220	0.8955
23	Akisik et al[39]	2011	Turkey	Caucasian	Hospital based	125	20	2	147	62	57	1	120	0.002*
24	Romanowicz et al[40]	2012	Poland	Caucasian	Hospital based	160	104	526	790	208	426	164	798	0.0451*
25	Krivokuca et al[41]	2013	Serbia	Caucasian	Hospital based	128	18	9	155	100	13	1	114	0.4401
26	Hosseini et al[42]	2013	Turkey	Caucasian	Hospital based	203	77	14	294	252	42	21	315	0.0000*
27	Smolarz et al[43]	2013	Poland	Caucasian	Hospital based	8	8	34	50	14	26	10	50	0.7412
28	Wasson et al[1]	2014	India	East Asian	Hospital based	117	76	12	205	134	73	10	217	0.9885
									**21236**				**19407**	

* Indicates statistical significance
